# Integrating single-cell and bulk RNA sequencing with ML algorithms identified MYBL2 as a candidate transcriptomic biomarker for diagnosing laryngeal squamous cell carcinoma (LSCC)

**DOI:** 10.1016/j.tranon.2026.103095

**Published:** 2026-11-01

**Authors:** Tahmineh Mehrabi, Marzie Isavi, Ali Gholami, Amir Ali Hamidieh, Roozbeh Heidarzadeh-Pilehrood

**Affiliations:** a Department of Cellular and Molecular Biology, Faculty of Advanced Science and Technology, Tehran Medical Sciences, Islamic Azad University, Tehran, Iran; b Department of Biological Sciences, Bowling Green State University, Bowling Green, OH, United States; c Department of Electrical Engineering, Faculty of Technology and Engineering, Central Tehran Branch, Islamic Azad University, Tehran, Iran; d Pediatric Cell and Gene Therapy Research Center, Gene, Cell & Tissue Research Institute, Tehran University of Medical Sciences, Tehran, Iran; e Department of Obstetrics & Gynecology, Faculty of Medicine and Health Sciences, Universiti Putra Malaysia, Serdang, 43400, Malaysia

**Keywords:** Laryngeal squamous cell carcinoma, MYBL2, Candidate transcriptomic biomarker, Machine learning, scRNA-seq, Malignant heterogeneity, Pseudotime analysis, Cell–cell communication

## Abstract

Laryngeal squamous cell carcinoma (LSCC) poses significant clinical challenges due to pronounced tumor heterogeneity. To identify robust, consistent biomarkers across cohorts, we integrated bulk RNA sequencing (RNA-seq) data from GSE127165 and GSE142083 and validated candidate biomarkers in the external cohort GSE13060. Candidate gene prioritization incorporated differentially expressed genes, weighted gene co-expression network analysis (WGCNA) results, and markers of malignant epithelial cells exhibiting high copy number variation (High-CNV) derived from single-cell RNA sequencing (scRNA-seq) (GSE206332). Additional analyses included functional enrichment, immune association (adjusted for tumor purity), quasi-temporal analysis, and CellChat inference to elucidate the biological roles of top genes. The integrated approach identified 254 differentially expressed genes (DEGs), comprising 86 upregulated and 168 downregulated genes. WGCNA revealed a tumor-associated module containing 870 genes. Intersecting these findings with high-CNV malignant epithelial markers reduced the candidate pool to 16 genes, and LASSO regression retained five key features: MYBL2, KRT17, UCHL1, PTHLH, and ODC1. Among these, MYBL2 demonstrated the strongest and most consistent performance. MYBL2 was further associated with proliferative programs, including E2F targets, the G2/M checkpoint, DNA repair, and MYC targets. Gene signatures from these subclusters were enriched in bulk tumors and positively correlated with MYBL2 expression in independent cohorts. The results also indicate that MYBL2-high malignant epithelial states are linked to distinct connectivity patterns within the tumor microenvironment.

## Highlights

• Bulk and single-cell RNA-seq identified 16 candidate diagnostic genes.
• MYBL2 showed robust diagnostic accuracy for LSCC across three datasets.
• MYBL2 marked a high-CNV malignant epithelial subcluster in LSCC.
• MYBL2-positive tumors linked to proliferation, DNA repair, and low immunity.
• Cell-cell communication implicated MDK, MIF, and SPP1 signaling in LSCC.

## Introduction

Laryngeal squamous cell carcinoma (LSCC) is one of the most important head and neck malignancies; it can also severely impair basic functions, including speech, swallowing, and airway maintenance [1]. According to global estimates (2022), approximately 189,200 new cases of laryngeal cancer and 103,359 deaths from the disease occurred worldwide [2]. Although advances in surgical methods, radiotherapy, chemotherapy, targeted therapy, and immunotherapy have improved management for some patients, clinical outcomes for those with advanced, recurrent, or refractory disease remain unfavorable. This situation largely reflects the significant heterogeneity of LSCC across genomic, transcriptional, cellular, and microenvironmental levels; thus, tumors with similar histological diagnoses may exhibit markedly different behaviors in proliferation rate, invasiveness, metastatic potential, response to therapy, and risk of recurrence. Therefore, identifying markers that more accurately reflect tumor biology and high-risk subpopulations remains a high priority in LSCC translational research [3,4].

Several molecular and histological markers are used to evaluate head and neck cancers and guide therapeutic decisions, but none alone fully captures the biological complexity of LSCC. p16 has diagnostic and prognostic significance in human papillomavirus (HPV)-associated tumors, especially in the oropharynx; however, its value in laryngeal cancer depends heavily on tumor location, actual HPV status, and the standard testing method [4,5]. Epidermal growth factor receptor (EGFR) is overexpressed in a significant proportion of head and neck squamous cell carcinoma cases. This overexpression has supported inhibitors such as cetuximab, but EGFR expression does not necessarily correlate directly with treatment response or tumor behavior at the individual level. Ki-67 also provides information about the cell cycle as an indicator of proliferative activity. Interpretation depends on sample type, intratumoral heterogeneity, staining method, and assessment threshold. In addition, markers such as p53 and cyclin D1, as well as alterations in genes related to the phosphoinositide 3-kinase/protein kinase B (PI3K/AKT), EGFR, and cell-cycle pathways, have been investigated in LSCC studies, but their independent and generalizable utility for distinguishing malignant, proliferative, and microenvironment-related states remains limited [6,7].

Bulk RNA-seq data can identify gene expression changes, dominant transcriptional programs, and co-expression modules associated with tumor phenotype; however, the resulting signal is a composite of multiple cell types and may obscure the characteristics of rare subpopulations or distinct malignant states. In contrast, scRNA-seq can more clearly reveal cellular heterogeneity, malignant epithelial populations, proliferation patterns, and predicted interactions between tumor cells and immune and stromal components [8,9]. From this perspective, integrating bulk and single-cell data with discriminant analysis, co-expression networks, feature selection methods, and machine learning (ML) algorithms can provide a systematic framework to reduce platform-specific bias and identify markers relevant to tumor cell biology.

Accordingly, in this study, we aim to identify and prioritize candidate transcriptomic markers associated with malignant heterogeneity and a proliferative phenotype in LSCC using ML algorithms applied to bulk and scRNA-seq datasets. We validate their predictive performance in independent cohorts, assess their association with cell cycle-related transcriptional programs, and investigate their relationship with cell subpopulations and tumor-microenvironment interactions.

## Materials and methods

### Data acquisition and preprocessing

We obtained LSCC expression datasets from the Gene Expression Omnibus (GEO) database (https://www.ncbi.nlm.nih.gov/geo/). We searched GEO using the term “Laryngeal Squamous Cell Carcinoma” and selected human high-throughput RNA-seq datasets that included both LSCC tumor tissues and adjacent normal mucosa samples. We combined GSE127165 (https://www.ncbi.nlm.nih.gov/geo/query/acc.cgi?acc=GSE127165) and GSE142083 (https://www.ncbi.nlm.nih.gov/geo/query/acc.cgi?acc=GSE142083) as the development cohort and retained GSE130605 (https://www.ncbi.nlm.nih.gov/geo/query/acc.cgi?acc=GSE130605) as an independent external test cohort. After preprocessing, we integrated the two development datasets using their shared genes. We matched the resulting expression matrix with the corresponding phenotype information and removed samples lacking complete expression or clinical-label data.

To avoid class imbalance, we randomly selected an equal number of tumor and normal samples from the merged cohort [10]. We then randomly split the balanced dataset into training and validation subsets at approximately a 7:3 ratio. We constructed the primary cohort using seed 123. We repeated cohort balancing and the approximately 7:3 training–validation split with seeds 1, 123, 456, and 999, while fixing model-fitting randomness to seed 123 (Supplementary Fig. S1). We used the training set to screen candidate transcriptomic biomarkers, the validation set to confirm internal performance, and the independent cohort to evaluate external tumor–normal classification performance.

### Differentially expressed gene (DEG) analysis

We assessed differential expression in the training cohort using linear models in limma (version 3.62.2) with empirical-Bayes moderation. We defined DEGs as those with Benjamini–Hochberg-adjusted P < 0.01 and |log fold change (logFC)| > 2. change (logFC)| > 2 [11].

### Weighted gene co-expression network analysis (WGCNA)

Weighted gene co-expression network analysis (WGCNA; package version 1.74) was applied to the 8000 genes with the highest median absolute deviation (MAD) [12,13]. We constructed a signed Pearson correlation network at β = 20 and transformed it into a topological overlap matrix (TOM). We grouped genes using average-linkage hierarchical clustering of TOM dissimilarity, and correlated module eigengenes with tumor status [14]. We retained genes with |module membership (MM)| ≥ 0.55 and |gene significance (GS)| ≥ 0.55 from the tumor-associated module. We repeated WGCNA for β = 12–20 to assess parameter sensitivity (Supplementary Fig. S2).

### Single-cell analysis and malignant marker identification

We analyzed three LSCC tumors from GSE206332 (GSM6251294, GSM6251297, and GSM6251300) using Seurat (version 5.5.0). We excluded cells with fewer than 200 detected genes or with at least 20% mitochondrial transcripts. The remaining cells were log-normalized, and 2000 variable genes and the first 30 principal components were used for principal component analysis (PCA), Harmony batch correction (version 1.2.4), graph-based clustering at a resolution of 0.8, and t-distributed stochastic neighbor embedding (t-SNE) visualization [15]. Cell types were assigned using published canonical markers, module scores, DotPlot visualization, and FindAllMarkers [16].

We performed an initial inferCNV analysis for each sample separately, using myeloid cells as the reference population. We calculated copy number variation (CNV) scores using an operational, data-driven criterion, taking the mean absolute deviation of inferCNV expression values from 1. We classified epithelial cells with scores exceeding the sample-specific 95th percentile of myeloid-reference scores as malignant-like.

We then reprocessed the malignant-like epithelial cells using LogNormalize normalization, 2000 variable genes, principal component analysis, and graph-based re-clustering at a resolution of 0.5 using the first 30 principal components, followed by t-SNE visualization. Low copy number variation (Low-CNV) internal reference subclusters were selected using a separate, data-driven operational criterion. We designated subclusters with median initial CNV scores at or below the 25th percentile as Low-CNV references. We capped reference selection at 35% of malignant subclusters; if we obtained fewer than 10 reference cells, we sequentially added subclusters with the next-lowest median CNV scores until we included at least 10 cells.

Refined inferCNV analysis [17]. We then performed inferCNV analysis using the selected Low-CNV subclusters as the internal reference population. After excluding the Low-CNV subclusters, we identified High-CNV marker genes with Seurat’s FindAllMarkers function using the Wilcoxon rank-sum test. We retained markers with avg_log2FC ≥ 2, min.pct ≥ 0.50, and Bonferroni-adjusted P < 0.001. We identified candidate genes by intersecting bulk upregulated DEGs, WGCNA-derived tumor-associated genes, and high-CNV markers. We assessed sensitivity to the marker definition using avg_log2FC thresholds of 1.5, 2.0, and 2.5, while keeping the remaining criteria fixed (Supplementary Fig. S3).

### Candidate transcriptomic biomarker identification based on tumor-normal discrimination

Overlapping candidate genes were evaluated using LASSO logistic regression with glmnet (version 4.1.10) [18]. We used a binomial model with tenfold cross-validation and retained genes with non-zero coefficients at lambda.min. We evaluated LASSO-selected genes using receiver operating characteristic (ROC) analysis with pROC. (Version 1.19.0.1) [19]. We used an area under the receiver operating characteristic curve (AUROC) ≥ 0.95 in both the training and validation cohorts as a stringent operational criterion for candidate prioritization.

We fitted a logistic regression panel of candidate genes in the training cohort after training-derived standardization and applied it to the validation and external cohorts without retraining. We compared the panel and MYBL2 using AUROCs with 95% confidence intervals and paired two-sided DeLong tests (Supplementary Fig. S4; Supplementary Table S1). We benchmarked MYBL2 against CDKN2A (p16), EGFR, and MKI67 (Ki-67) using cohort-specific AUROCs and paired two-sided DeLong tests; we fixed the ROC direction from the training cohort (Supplementary Fig. S5). This sequential procedure served as a candidate-prioritization strategy rather than a series of confirmatory hypothesis tests, and we reserved the independent GSE130605 cohort exclusively for external evaluation.

### External validation using ML algorithms

We further evaluated candidate genes using RF and SVM classifiers trained on the training cohort and tested on GSE130605. We applied training-derived standardization externally. RF used 500 trees with randomForest (version 4.7–1.2), whereas SVM used a linear kernel with e1071 (version 1.7–17) [20,21]. We calculated classification metrics from default-class predictions, while we computed AUROC from predicted tumor probabilities. We used the validation cohort solely for internal biomarker assessment. We summarized performance using AUROC, accuracy, sensitivity, specificity, precision, and F1-score. We compared RF and SVM AUROCs on the same external samples using a paired two-sided DeLong test.

### Immune microenvironment analysis

We used pooled tumors from GSE127165 and GSE142083 for immune analyses. We used median-split MYBL2 groups descriptively, whereas the primary analysis treated MYBL2 as a continuous variable. We estimated immune-cell enrichment by single-sample gene set enrichment analysis (ssGSEA) using gene set variation analysis (GSVA) (version 2.0.7) and 28 published signatures [22,23]. We tested group differences with Wilcoxon tests with Benjamini–Hochberg correction; unadjusted Spearman correlations were descriptive.

We estimated stromal and immune cell proportions in malignant tumor tissues using Expression data (ESTIMATE), and calculated purity scores using Oncology Biological Research (IOBR) (version 2.2.3) and the published ESTIMATE transformation [24,25]. We compared stromal, immune, and ESTIMATE scores between the MYBL2-high and MYBL2-low groups using Wilcoxon rank-sum tests, with Benjamini–Hochberg correction applied across the three comparisons. We assessed primary immune associations using partial Spearman correlations between continuous MYBL2 and the 28 immune scores, controlling for estimated purity; we defined significance as Benjamini–Hochberg false discovery rate (BH-FDR) < 0.05. Sensitivity analyses also controlled for dataset identity (Supplementary Table S2).

### MYBL2 and functional programs in high-CNV malignant subclusters

We analyzed previously defined High-CNV subclusters without re-clustering, using the existing t-SNE embedding. DotPlot summarized MYBL2, and program activity was calculated from mean gene-wise Z-scores for Hallmark and curated signatures [26]. The Cluster 0–Cluster 8 program comparison was descriptive; no inferential tests were applied.

### Bulk-level validation of selected high-CNV malignant subcluster signatures and their association with MYBL2

#### ssGSEA of selected high-CNV malignant subcluster signatures

We derived Cluster 0 and Cluster 8 signatures from strict High-CNV markers after excluding MYBL2, mitochondrial, ribosomal, and shared genes; we retained up to 50 top-ranked markers per signature. We calculated bulk signature scores using GSVA and compared them between tumors and normal samples using Wilcoxon tests with Benjamini–Hochberg correction [23]. We used Spearman correlations to relate MYBL2 to both signature scores in discovery-cohort tumors; we applied Benjamini–Hochberg correction to the two P-values.

#### MYBL2-continuous (GSEA) in bulk LSCC tumors

For GSEA, we ranked genes by their continuous association with standardized MYBL2 in discovery-cohort tumors using limma, with dataset identity as a covariate; we excluded MYBL2 itself. Pre-ranked GSEA used fgsea (version 1.32.4) and the Molecular Signatures Database (MSigDB) Hallmark sets [26,27], with adjusted *P* < 0.05 defining significance.

### Pseudotime analysis of high-CNV malignant epithelial subclusters

We ordered high-CNV subclusters using Monocle 2 (version 2.34.0) and the Discriminative dimensionality reduction tree (DDRTree) and selected subcluster markers. We defined pseudotime as the graph distance along the inferred principal tree, rooted at the state with the lowest median G2/M-related score. We visualized MYBL2 patterns in Clusters 0 and 8 with locally estimated scatterplot smoothing (LOESS) curves and approximate 95% confidence bands [28,29].

### Cell–cell communication analysis

We inferred putative ligand–receptor interactions with CellChat (version 2.2.0.9001) and human CellChatDB. We treated high-CNV subclusters and major microenvironment populations as distinct groups. We estimated outgoing patterns for Clusters 0 and 8 with triMean, requiring ≥15 cells per group and nominal *P* < 0.05.

## Results

### Dataset preprocessing and cohort construction

Fig. 1 and Table 1 summarize the study workflow and dataset characteristics. The merged development cohort contained 219 samples; after balancing, it included 218 (109 tumors and 109 normal samples). We divided the balanced cohort into training (n = 154; 77 tumors and 77 normal samples) and validation (n = 64; 32 tumors and 32 normal samples) sets; GSE130605 served as the independent external cohort. We excluded only one sample during balancing, and performance varied little across seeds 1, 123, 456, and 999 (Supplementary Fig. S1).

**Fig. 1:**
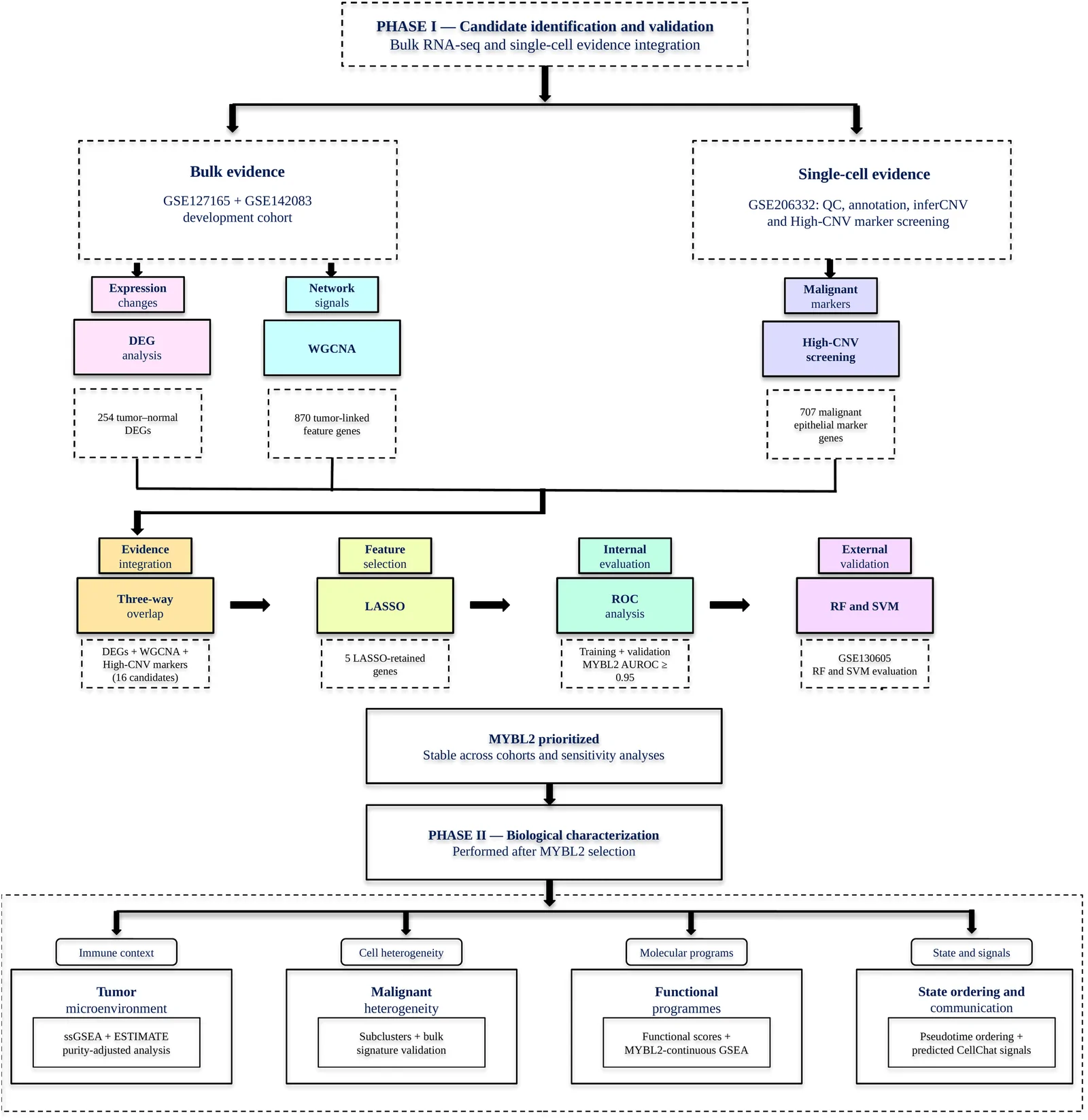
Study workflow delineating the identification and characterization of MYBL2-associated malignant heterogeneity in LSCC. Public bulk and single-cell transcriptomic datasets were integrated into a biphasic analytical framework. In Phase I, candidate genes were pinpointed employing differential expression analysis, weighted gene co-expression network analysis, and inferCNV-based screening for malignant epithelial markers. Overlapping candidates were prioritized via LASSO regression, receiver operating characteristic analysis, and external validation utilizing random forest and support vector machine models, ultimately leading to the selection of MYBL2. In Phase II, MYBL2-associated biological features were systematically characterized through analysis of the immune microenvironment, malignant cell subcluster profiling, functional enrichment analysis, pseudotime inference, and the assessment of predicted cell–cell communication.

**Table 1:** Summary of RNA-seq and single-cell RNA-seq datasets.

GEO accession	Platform	Samples	Attribute
GSE127165	GPL20301	57 Normal / 57 LSCC	Training and validation set
GSE142083	GPL20301	53 Normal / 53 LSCC	Training and validation set
GSE130605	GPL20301	50 Normal / 50 LSCC	Independent test set
GSE206332	GPL18573	3 LSCC	Single-cell RNA-seq set

### DEG screening

We performed DEG analysis on the training set to identify genes that differed significantly between LSCC tumor and normal samples. Using the predefined thresholds of |logFC| > 2 and an adjusted *P*-value < 0.01, we identified 254 DEGs, including 86 upregulated and 168 downregulated genes. The volcano plot shows their overall distribution, and the heatmap presents the expression patterns of all significant DEGs (Fig. 2a–b).

**Fig. 2:**
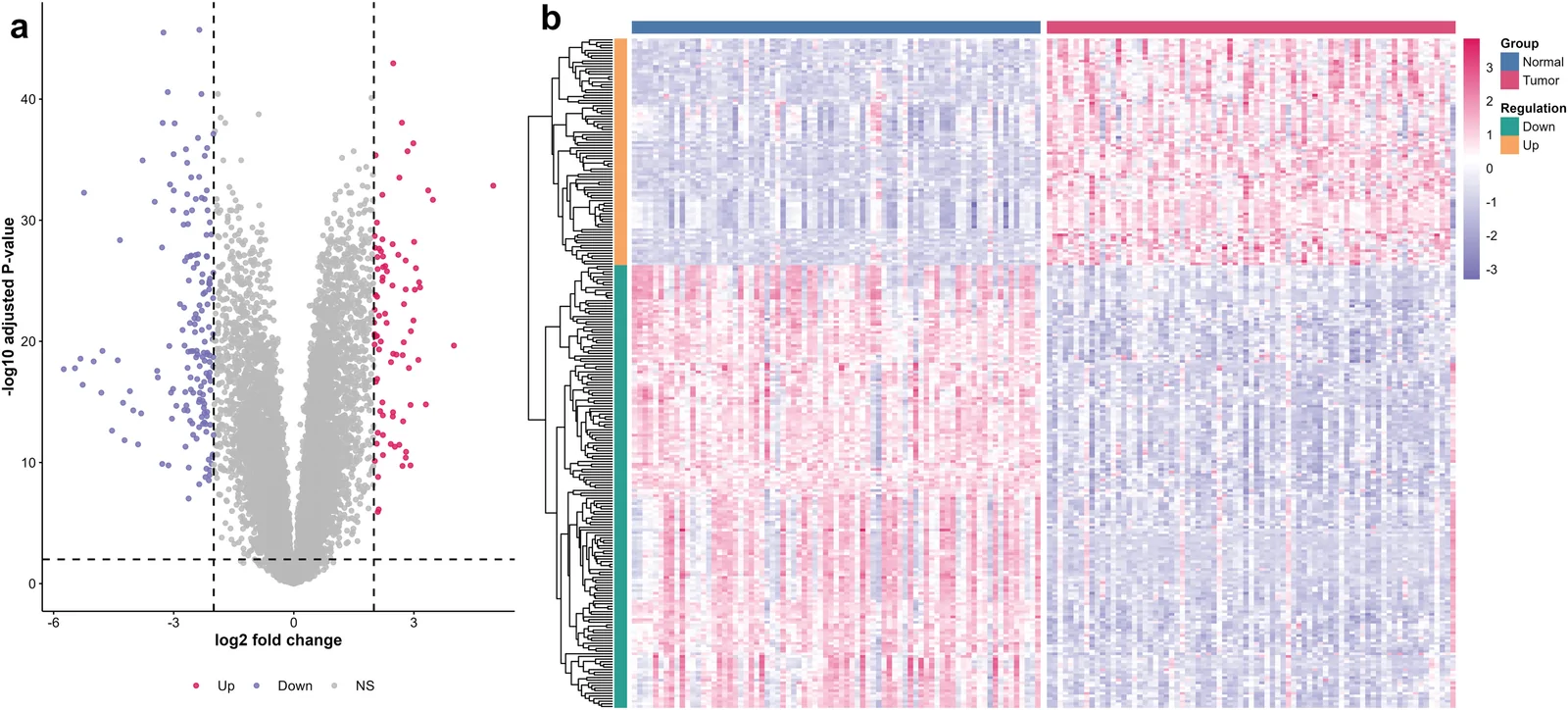
Differential Expression Analysis in the Training Cohort. (a) Volcano plot of differentially expressed genes (DEGs) comparing LSCC tumor samples and normal samples. (b) Heatmap illustrating expression profiles of significant DEGs across normal and LSCC tumor samples. Here, "n" represents individual bulk-tissue samples within the training cohort (n = 154: 77 tumor samples and 77 normal samples). Differential expression was evaluated using limma linear models with empirical-Bayes moderation; DEGs were considered significant if they demonstrated an absolute log fold change greater than 2 and a Benjamini-Hochberg-adjusted P-value <0.01.

### WGCNA analysis of the LSCC training dataset

To complement DEG screening and identify co-expression modules and genes associated with LSCC tumor status, we subsequently performed WGCNA on the training cohort. The cohort comprised 154 samples, with equal numbers of normal and tumor samples (Fig. 3a). A soft-thresholding power of β = 20 was selected to construct the signed co-expression network, yielding a signed scale-free topology fit of 0.8918 (Fig. 3b). Using this network, 15 non-gray co-expression modules were identified (Fig. 3c).

**Fig. 3:**
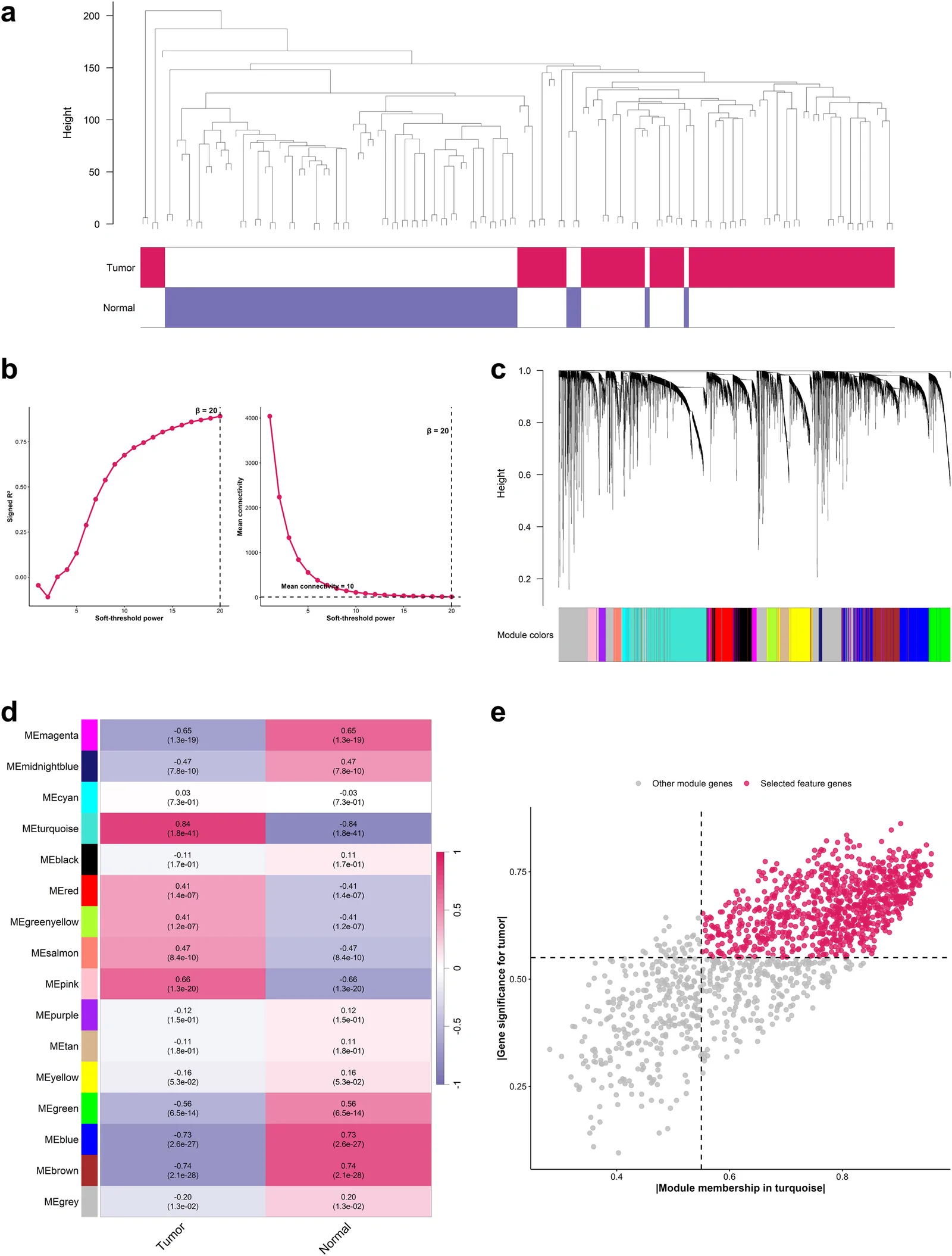
WGCNA analysis of the training cohort. (a) Sample dendrogram and trait heatmap of the training cohort. (b) A soft-thresholding power of β = 20 (signed R² = 0.891) was selected based on the scale-free topology fit and mean connectivity. (c) Hierarchical clustering dendrogram of genes with corresponding module color assignments. (d) Heatmap illustrating relationships between module eigengenes and tumor/normal phenotypes. Each row denotes a module, and each column denotes a phenotype; each cell displays the correlation coefficient and the corresponding P-value. (e) Scatter plot depicting absolute gene significance for tumor status versus absolute module membership in the turquoise module. Module-trait and gene-level associations were assessed using Pearson correlation, with relevant P-values indicated where applicable. Here, n signifies individual bulk-tissue samples (n = 154: 77 tumors and 77 normal samples). WGCNA feature genes were preserved using criteria of |MM| ≥ 0.55 and |GS| ≥ 0.55.

Module–trait correlation analysis showed that the turquoise module had the strongest positive association with LSCC tumor status (r = 0.8361, P = 1.82 × 10⁻⁴¹). In contrast, the brown module showed the strongest negative association (r = −0.7439, P = 2.12 × 10⁻²⁸) (Fig. 3d). Because the turquoise module showed the strongest positive correlation with the tumor phenotype, we selected it for further analysis. Within the turquoise module, MM and GS were strongly and positively correlated (r = 0.7903, P = 8.12 × 10⁻³⁰⁸) (Fig. 3e), indicating that highly connected genes in this module were also strongly associated with LSCC tumor status. Applying the thresholds |MM| ≥ 0.55 and |GS| ≥ 0.55 identified 870 WGCNA-derived tumor-associated feature genes for downstream analyses. Across β = 12–20, the principal module–tumor association remained stable, and MYBL2 was retained at every tested power (Supplementary Fig. S2).

### Single-cell and malignant marker results

To add cell-type-specific evidence to the biomarker-screening strategy, we performed single-cell analysis to characterize LSCC cellular heterogeneity and identify marker genes associated with High-CNV malignant epithelial cells. We analyzed scRNA-seq data with Seurat. After quality control, normalization, and clustering, 30 cell clusters were identified and visualized with t-SNE (Fig. 4a). Based on canonical marker genes, cells were classified into major cell populations, including epithelial cells, T cells, B cells, myeloid cells, natural killer (NK) cells, endothelial cells, and fibroblasts (Fig. 4b).

**Fig. 4:**
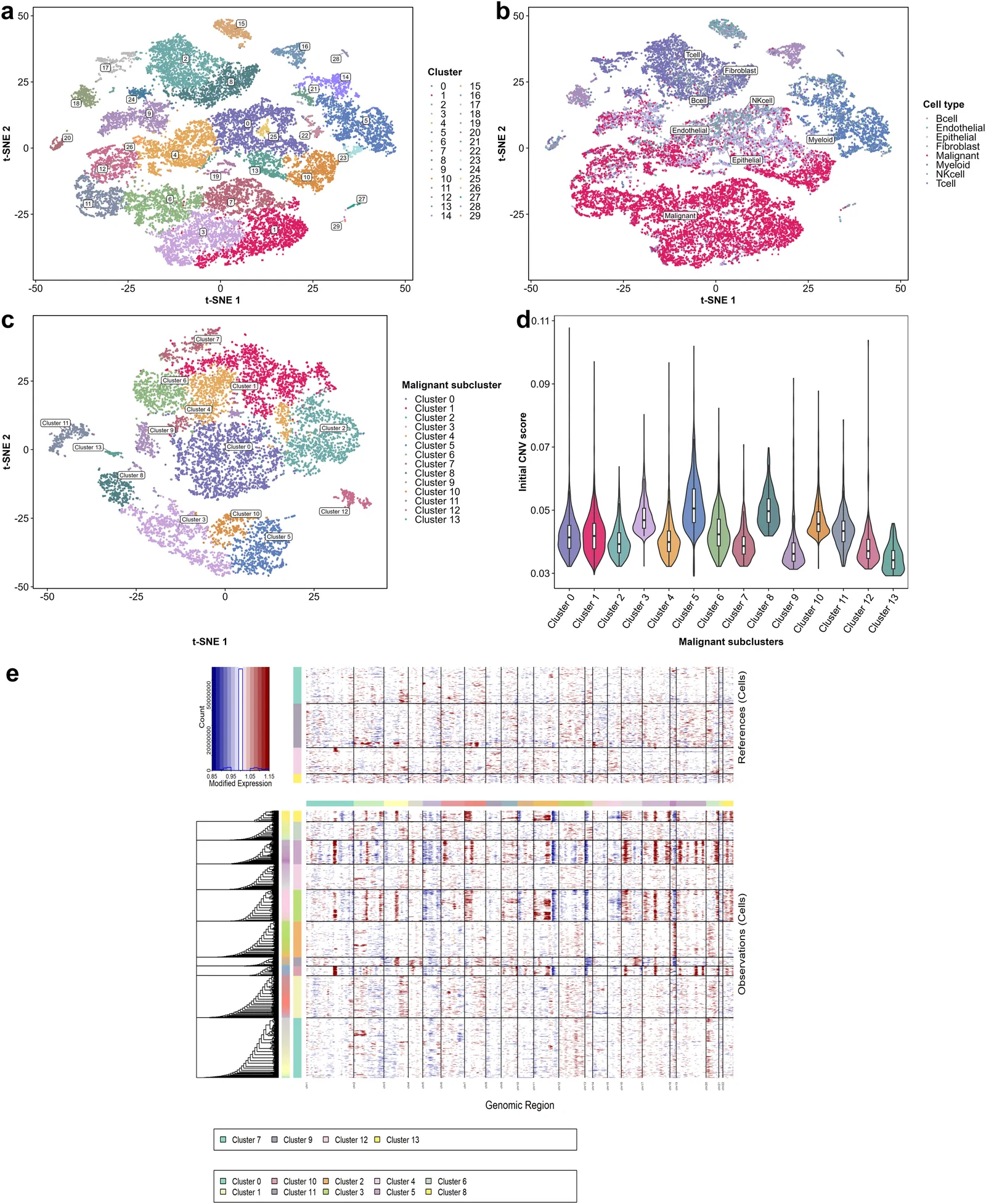
Single-cell RNA sequencing analysis and copy number variation (CNV)-based characterization of malignant-like epithelial cells in LSCC. (a) t-SNE visualization illustrating the global cell clusters. (b) Annotation of principal cell populations, including malignant-like epithelial cells. (c) Re-clustering of malignant-like epithelial cells into subclusters of malignant epithelial cells. (d) Distribution of initial CNV scores employed to identify Low-CNV malignant subclusters as internal reference groups for the refined inferCNV analysis. (e) Refined inferCNV heatmap depicting extensive chromosomal CNV patterns across malignant epithelial subclusters. We obtained data from three independent LSCC tumors (GSM6251294, GSM6251297, and GSM6251300). For the single-cell panels, the analytical unit is the individual cell, whereas the biological sampling unit is the patient tumor.

B cells were characterized by the expression of CD79A, CD19, and CD79B; endothelial cells by KDR, FLT1, TEK, and ICAM1; epithelial cells by EPCAM, KRT15, KRT18, and KRT19; fibroblasts by ACTA2, FAP, and S100A4; myeloid cells by CD68, CD33, CD1E, LYZ, and LAMP3; NK cells by NCAM1, FCGR3A, NCR1, and NCR3; and T cells by CD2, CD3D, CD3E, and CD3G. We identified malignant-like epithelial cells using an operational, data-driven criterion: epithelial cells with CNV scores exceeding the sample-specific 95th percentile of the corresponding myeloid-reference scores were classified as malignant-like. We isolated these cells and re-clustered them into 14 malignant epithelial subclusters to further assess intratumoral heterogeneity (Fig. 4c). Using this data-driven 25th-percentile criterion based on cluster-level median initial CNV scores, four subclusters (Clusters 7, 9, 12, and 13) met the Low-CNV criterion and were used as internal reference groups for the refined inferCNV analysis (Fig. 4d).

The refined analysis revealed broad chromosomal CNV alterations across malignant epithelial cells. After removing the four Low-CNV subclusters, we classified the remaining 10 subclusters as High-CNV malignant epithelial subclusters (Fig. 4e). We then performed marker analysis across these High-CNV malignant epithelial subclusters. Using thresholds of avg_log2FC ≥ 2, min.pct ≥ 0.50, and a Bonferroni-adjusted P-value < 0.001, we identified 707 High-CNV malignant epithelial marker genes.

### Screening for candidate transcriptomic biomarkers

The intersection of bulk-upregulated DEGs, WGCNA tumor-associated genes, and high-CNV markers yielded 16 candidates (Fig. 5a). LASSO retained MYBL2, KRT17, UCHL1, PTHLH, and ODC1 at lambda.min (Fig. 5b). MYBL2 was the only retained gene with AUROC ≥ 0.95 in both the training (0.9712) and validation (0.9658) cohorts (Fig. 5c). MYBL2 expression was also higher in tumors in both cohorts (both P < 0.001; Fig. 5d). MYBL2 was retained across permissive, primary, and stringent marker definitions (Supplementary Fig. S3). The five-gene panel did not consistently outperform MYBL2 alone across the three cohorts (Supplementary Fig. S4; Supplementary Table S1). Supplementary Fig. S5 reports cohort-specific ROC curves, AUROCs, and paired two-sided DeLong comparisons of MYBL2 with transcript-level p16, EGFR, and Ki-67.

**Fig. 5:**
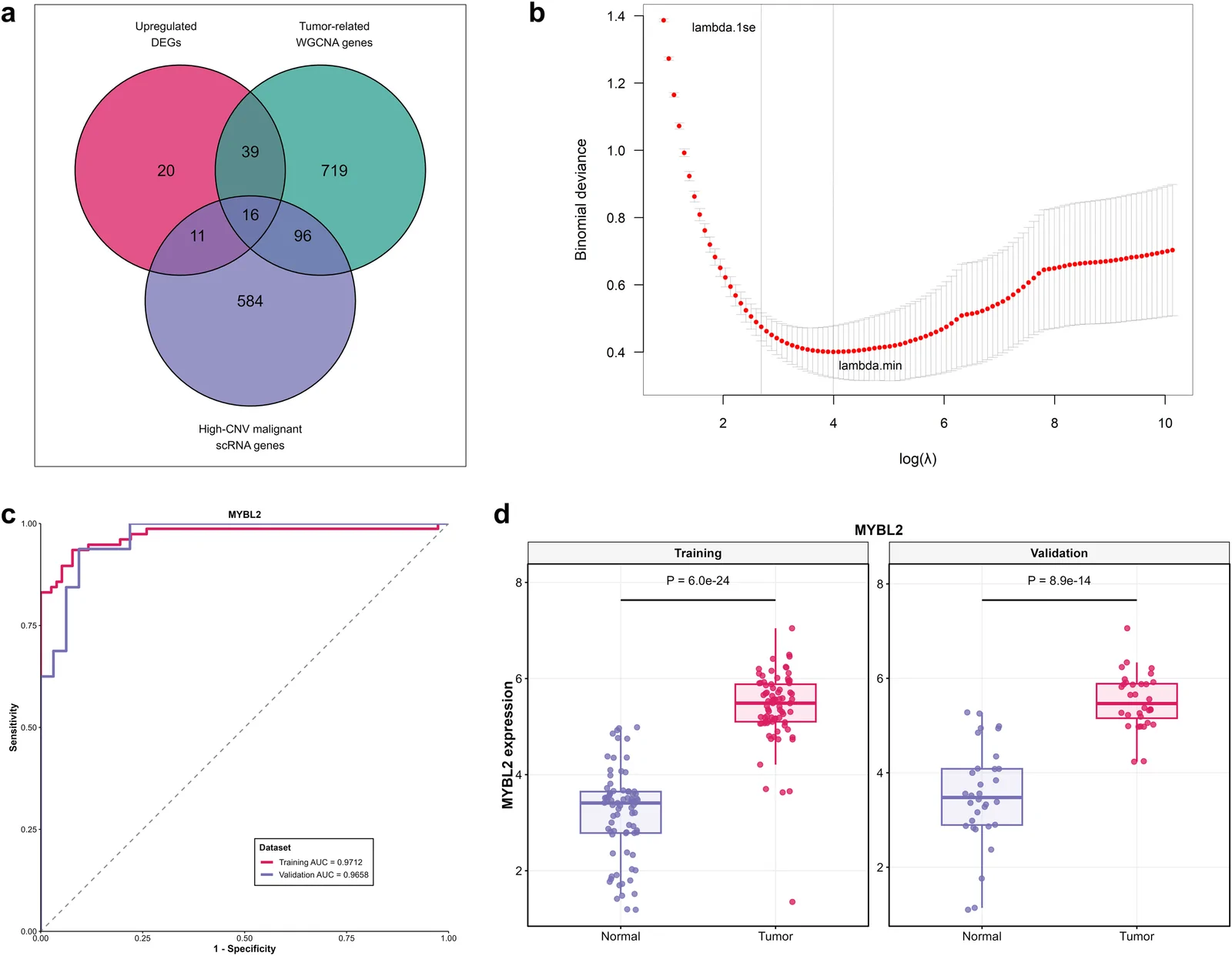
Evaluation and internal validation of MYBL2 as a potential transcriptomic biomarker for distinguishing tumor and normal tissues in LSCC. (a) Venn diagram showing the intersection of genes upregulated in bulk RNA sequencing, tumor-associated feature genes identified by WGCNA, and high-CNV malignant epithelial marker genes, yielding 16 candidate genes. (b) LASSO cross-validation curve used for feature selection. (c) Receiver Operating Characteristic (ROC) curves for MYBL2 in the training and internal validation cohorts. (d) MYBL2 expression levels in normal versus LSCC tumor samples across the training and validation cohorts. Here, n indicates the number of bulk-tissue samples: training cohort, n = 154 (77 tumor and 77 normal samples); internal validation cohort, n = 64 (32 tumor and 32 normal samples).

### External validation using machine-learning classifiers

In GSE130605, both MYBL2-based classifiers discriminated tumors from normal samples (Table 2). SVM achieved AUROC 0.9624, accuracy 0.870, sensitivity 0.780, and specificity 0.960; RF achieved AUROC 0.9100, accuracy 0.810, sensitivity 0.740, and specificity 0.880. SVM had a higher AUROC than RF (difference = 0.0524; paired two-sided DeLong *P* = 0.0056; Fig. S1).

**Table 2:** External performance comparison of MYBL2-based machine learning classifiers in the GSE130605 cohort.

ML method	Dataset	AUC	Accuracy	Sensitivity	Specificity	Precision	F1-score
RF	GSE130605	0.9100	0.81	0.74	0.88	0.860	0.796
SVM	GSE130605	0.9624	0.87	0.78	0.96	0.951	0.857

### MYBL2-associated immune microenvironment

Unadjusted analyses showed lower enrichment of 15 immune signatures and lower stromal, immune, and ESTIMATE scores in MYBL2-high tumors (Fig. 6a–b). After purity adjustment, most negative associations were attenuated; NK T cells, regulatory T cells, and immature B cells remained significant (Fig. 6c; Supplementary Table S2). Additional adjustment for dataset identity yielded comparable results, indicating that the broad unadjusted negative pattern was not independent of tumor purity (Supplementary Table S2).

**Fig. 6:**
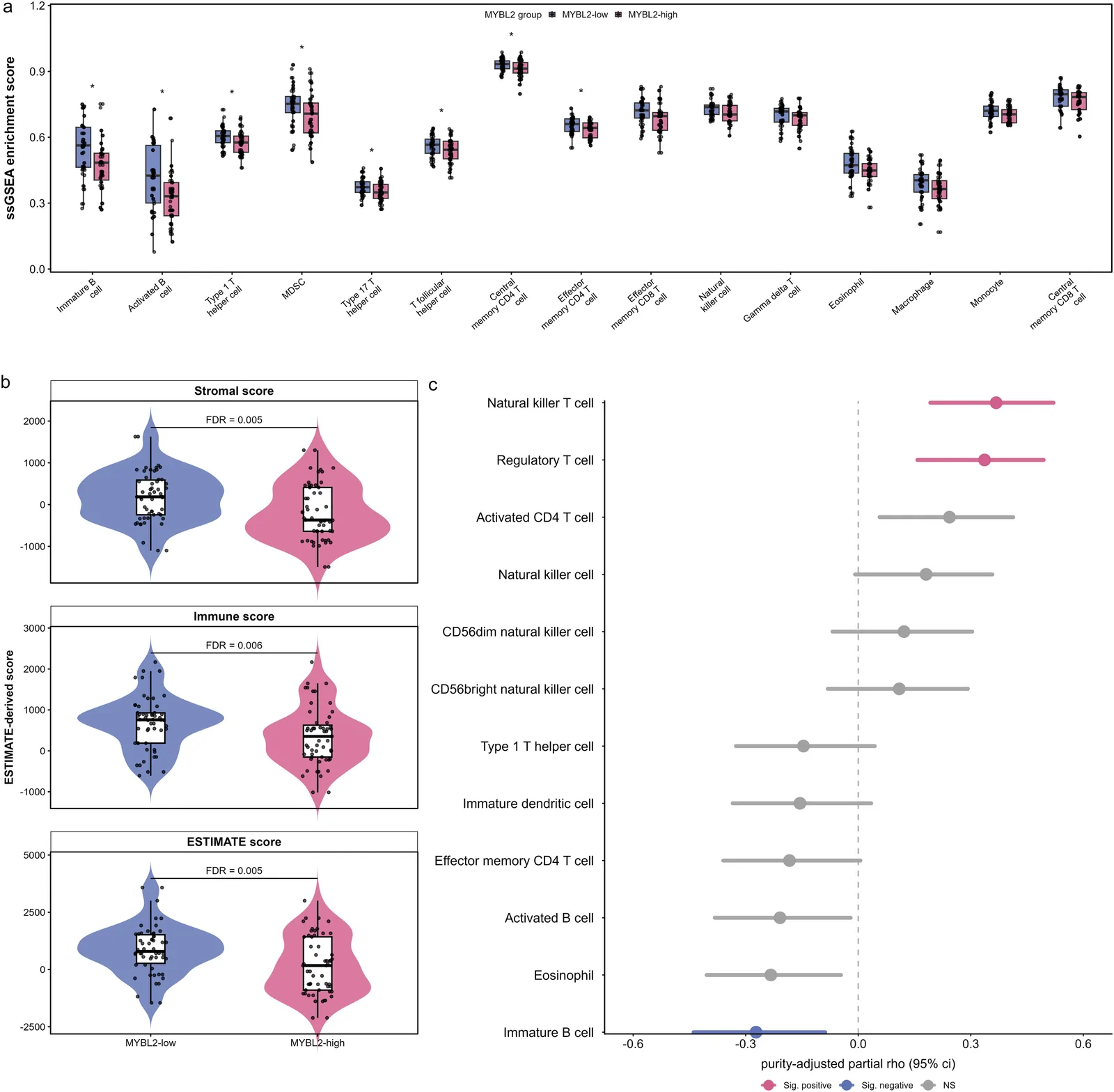
MYBL2-Associated immune microenvironment analysis in LSCC. (a) Unadjusted ssGSEA immune-cell enrichment comparisons between MYBL2-Low and MYBL2-High Tumors; asterisks indicate BH-FDR < 0.05. (b) Unadjusted Stromal, Immune, and ESTIMATE Scores in MYBL2-Low and MYBL2-High Tumors. (c) Tumor-Purity-Adjusted Partial Spearman Correlations between Continuous MYBL2 Expression and Immune-Cell Enrichment Scores. Points indicate partial rho values, and horizontal lines indicate approximate 95% confidence intervals; significant positive, significant negative, and non-significant associations are distinguished as shown in the legend. All 28 immune-cell signatures were tested with Benjamini-Hochberg correction, while the strongest 12 associations are displayed for compact visualization. In the immune analyses, N denotes individual LSCC bulk-tissue tumor samples from the pooled discovery cohort (N = 109). MYBL2-High versus MYBL2-Low comparisons used Wilcoxon rank-sum tests with Benjamini-Hochberg correction, and continuous immune associations used Spearman or tumor-purity-adjusted partial Spearman correlation, as specified in the methods. BH-FDR < 0.05 defined statistical significance across the corresponding 28 immune-cell tests.

### Characterization of high-CNV malignant epithelial subclusters and functional program activity

Ten High-CNV malignant epithelial subclusters exhibited transcriptional heterogeneity (Fig. 7a). Cluster 0 had the highest scaled average MYBL2 expression. In contrast, Cluster 8 had the highest proportion of expressing cells (Fig. 7b). Cluster 8 showed modestly higher activity in E2F-target, G2/M-checkpoint, DNA-repair, and stress-response pathways. In contrast, Cluster 0 showed modestly higher MYC-target, epithelial-differentiation, and senescence-like scores.

**Fig. 7:**
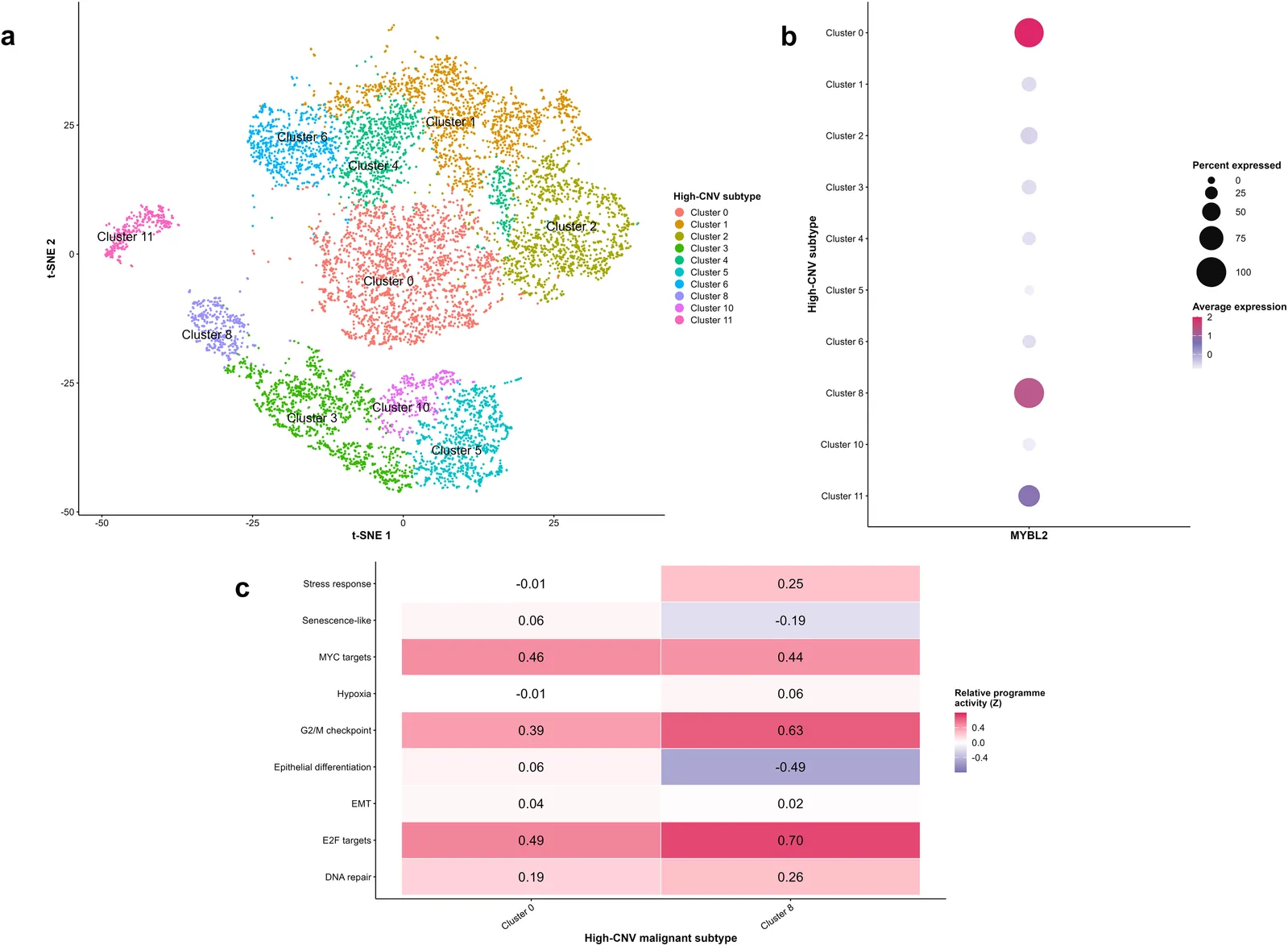
High-CNV malignant epithelial subclusters and MYBL2-associated functional programs. (a) t-SNE distribution of the ten retained High-CNV malignant epithelial subclusters. (b) MYBL2 DotPlot across subclusters; dot size indicates the percentage of MYBL2-expressing cells and color indicates scaled average expression. (c) Relative functional-program activity in Cluster 0 and Cluster 8, calculated as mean gene-wise Z-scores. Data were derived from three independent LSCC tumors. The analytical unit in panels a-b is the individual cell, whereas the biological sampling unit is the patient tumor. Panel c shows a descriptive subcluster-level summary of mean gene-wise Z-scores; no separate inferential hypothesis test was applied to the Cluster 0-Cluster 8 program-score comparison.

### Bulk-level validation of selected high-CNV malignant subcluster signatures and their association with MYBL2

#### Bulk-level validation of high-CNV malignant cluster 0 and cluster 8 signatures

Cluster 0 and Cluster 8 signatures were enriched in tumors in both the discovery and GSE130605 cohorts (all BH-FDR < 0.001; Fig. 8a–b). In discovery-cohort tumors, MYBL2 correlated positively with Cluster 0 (ρ = 0.64) and Cluster 8 (ρ = 0.66) signature scores (both BH-FDR < 0.001; Fig. 8c).

**Fig. 8:**
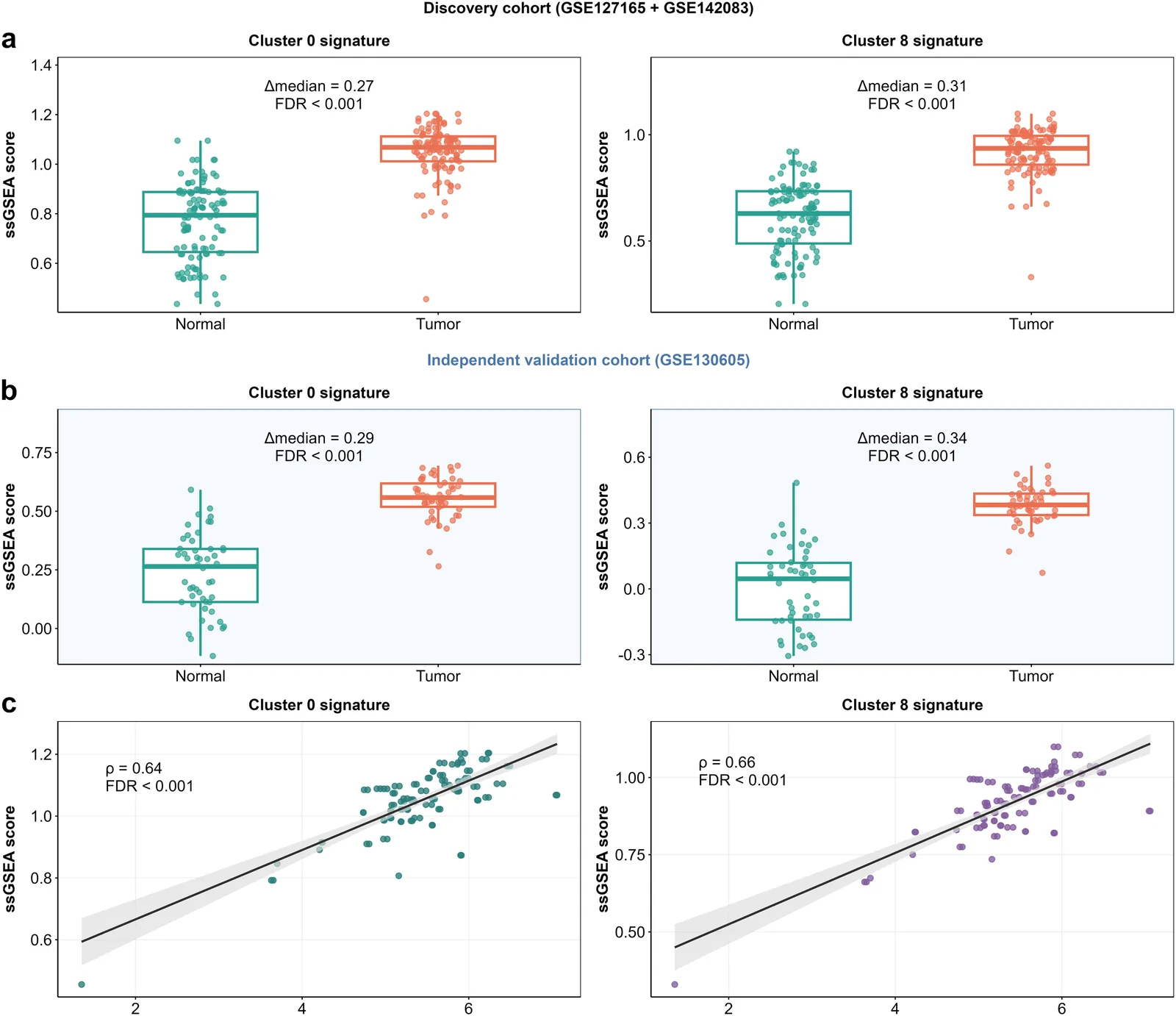
Bulk-level validation of High-CNV malignant Cluster 0 and Cluster 8 signatures in LSCC. (a) ssGSEA scores in normal and tumor samples from the combined discovery cohort. (b) Independent validation in GSE130605. (c) Associations between MYBL2 expression and the Cluster 0 or Cluster 8 signature score in discovery-cohort tumor samples. The analytical unit is the individual bulk-tissue sample. The combined discovery cohort comprised n = 218 samples (109 tumors and 109 normal samples), and the independent GSE130605 cohort comprised n = 100 samples (50 tumors and 50 normal samples). Panel c used the 109 discovery-cohort tumor samples. Tumor-normal comparisons used Wilcoxon rank-sum tests with Benjamini-Hochberg correction, while MYBL2-signature associations used Spearman correlation with Benjamini-Hochberg correction.

#### MYBL2-associated hallmark pathway enrichment in bulk LSCC tumors

Pre-ranked GSEA associated MYBL2 with E2F Targets normalized enrichment score (NES) = 4.21, G2/M Checkpoint (NES = 4.05), DNA Repair (NES = 2.58), and MYC Targets V1 NES = 3.59; all false discovery rate (FDR) < 0.001; Fig. 9).

**Fig. 9:**
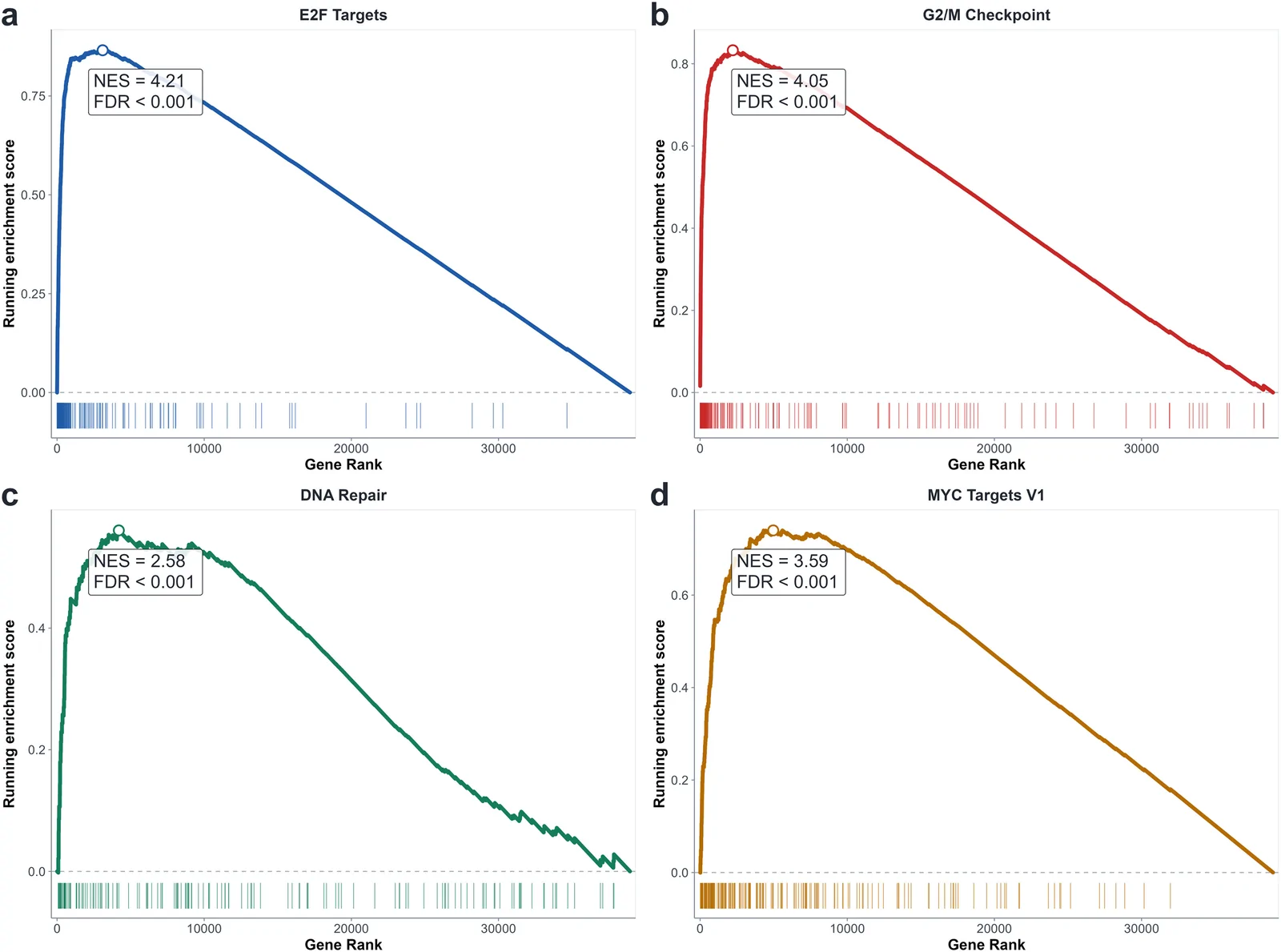
MYBL2-associated Hallmark pathway enrichment in bulk LSCC tumors. Pre-ranked GSEA showed significant positive enrichment of (a) E2F Targets, (b) G2/M Checkpoint, (c) DNA Repair, and (d) MYC Targets V1 among genes positively associated with MYBL2 expression (all FDR < 0.001). The analytical unit was the individual discovery-cohort tumor sample (n = 109). Genes were ranked using the moderated limma statistic for MYBL2 association, and pathway significance was assessed by pre-ranked GSEA with Benjamini-Hochberg adjustment; FDR < 0.05 was the predefined significance threshold.

### Pseudotime analysis of high-CNV malignant epithelial subclusters

DDRTree ordered the ten High-CNV subclusters across seven inferred transcriptional states, with State 1 as the root (Fig. 10a–c). In Cluster 0, MYBL2 increased and approached a plateau along the inferred ordering, whereas Cluster 8 was comparatively stable, with lower expression at higher pseudotime values (Fig. 10d–e).

**Fig. 10:**
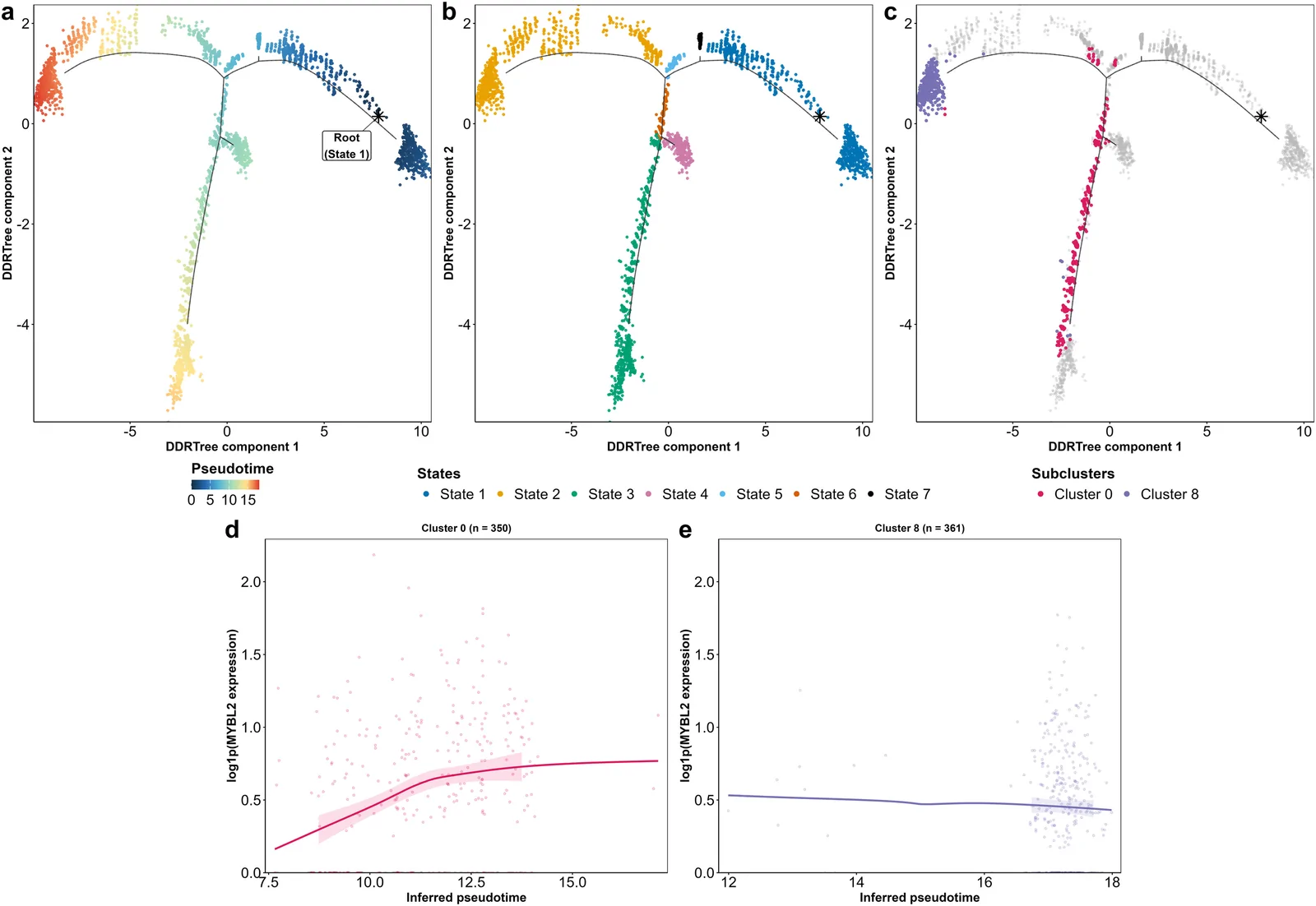
DDRTree-based graph pseudotime analysis of High-CNV malignant epithelial subclusters. (a) Cells are colored by inferred pseudotime and the State 1 root. (b) Distribution of seven inferred pseudotime states. (c) Clusters 0 and 8 are highlighted relative to the remaining subclusters. (d-e) Trends in MYBL2 expression across pseudotime in Clusters 0 and 8; lines denote LOESS fits, and shaded regions represent 95% confidence intervals. Data were obtained from three independent LSCC tumors. The analytical unit consists of individual High-CNV malignant epithelial cells, while the biological sampling unit is the patient tumor.

### Predicted cell–cell communication patterns of selected high-CNV malignant epithelial subclusters

CellChat predicted outgoing communication from Clusters 0 and 8 to immune and stromal populations, with myeloid cells among the strongest predicted recipients (Fig. 11). Top-ranked putative interactions involved MDK-, MIF-, and SPP1-related signaling, including the coreceptor complexes MDK–NCL, SPP1–CD44, and MIF–CD74.

**Fig. 11:**
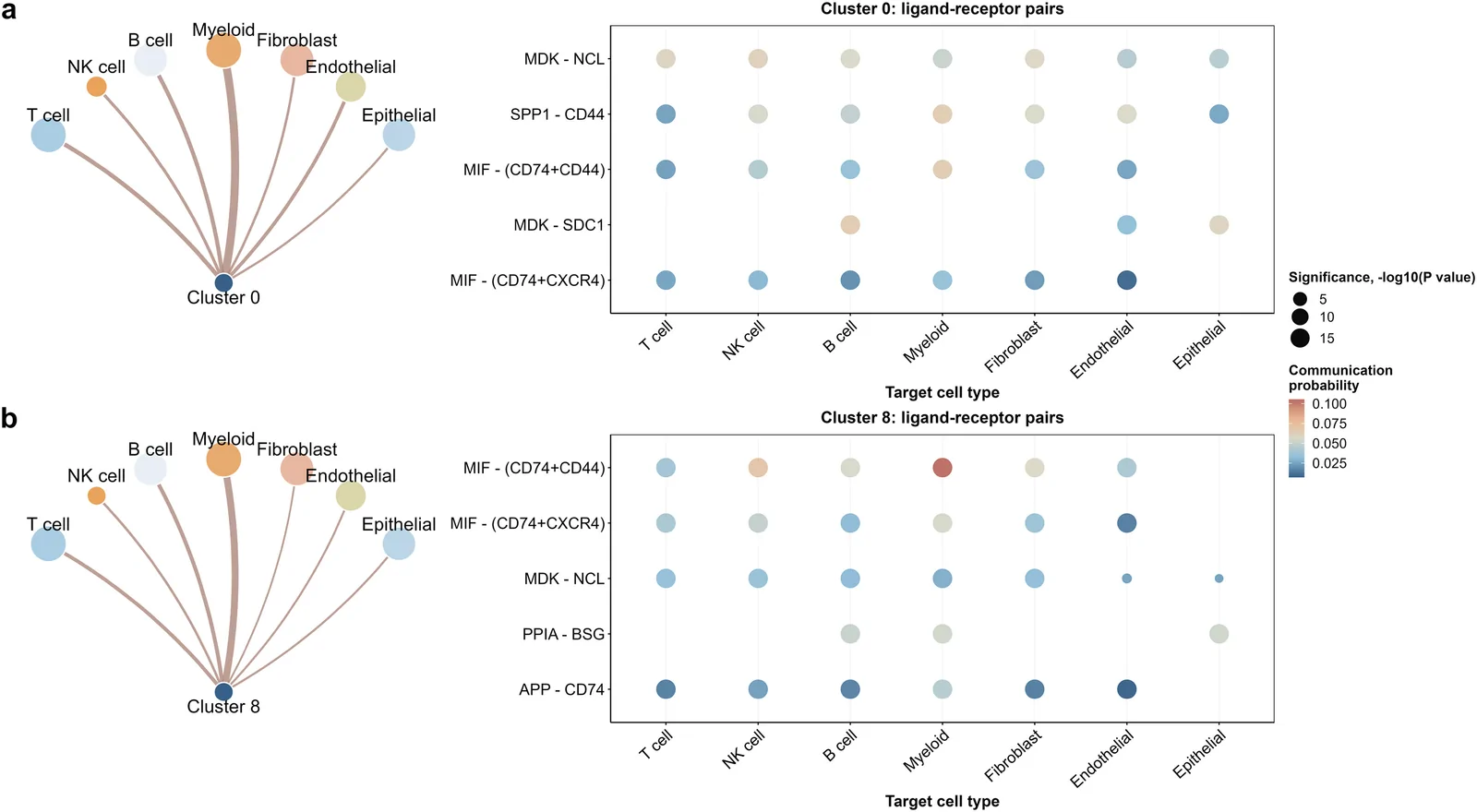
Predicted outgoing cell–cell communication in selected High-CNV malignant epithelial subclusters. (a) Outgoing communication network and top ligand–receptor pairs for Cluster 0. (b) Outgoing communication network and top ligand–receptor pairs for Cluster 8. Line width indicates inferred interaction weight; dot color indicates communication probability; dot size indicates statistical significance. Data were derived from three independent LSCC tumors. The analytical unit for CellChat input is the individual cell, whereas the biological sampling unit is the patient tumor; cell groups were required to contain at least 15 cells. CellChat interactions retained for visualization had nominal P < 0.05.

## Discussion

MYBL2 should be regarded as a candidate transcriptomic biomarker rather than a clinically validated diagnostic marker. In the present study, an integrative computational framework combining bulk RNA-seq, single-cell transcriptomics, WGCNA, and ML approaches identified MYBL2 as the most consistent candidate among the evaluated genes. MYBL2 showed reproducible tumor–normal discrimination across independent cohorts, whereas the five-gene panel did not consistently outperform MYBL2 alone. These findings suggest that, although multigene models may capture broader molecular information, individual genes representing core malignant biological processes may still demonstrate consistent classification performance. However, transcriptomic discrimination between established tumor tissues and adjacent normal mucosa should not be interpreted as equivalent to clinical utility. The absence of benign lesions, inflammatory conditions, and premalignant dysplastic tissues may overestimate tumor–normal discrimination performance. Therefore, prospective validation in clinically relevant comparator cohorts will be necessary before MYBL2 can be considered for clinical application.

The biological significance of MYBL2 in this study is underscored by its established functions in regulating cell-cycle progression, cell proliferation, DNA replication, and maintaining genomic stability. Also called B-Myb, MYBL2 is part of the MYB transcription factor family and is essential for the transition from S phase to mitosis, as it regulates genes associated with DNA synthesis and mitotic progression [30]. Dysregulation of MYBL2 has been reported across multiple cancer types, and increased expression is often linked to enhanced proliferative capacity, poor differentiation, and aggressive tumor characteristics. In LSCC, recent evidence suggests that MYBL2 may contribute to tumor progression by interacting with GTSE1 and activating PI3K/AKT-dependent glycolytic reprogramming, indicating that MYBL2 may function beyond a simple proliferation marker and participate in metabolic adaptation in malignant cells [31].

Consistent with previous observations, our analysis showed that MYBL2 expression was strongly associated with several proliferation-related transcriptional programs, including E2F targets, G2/M checkpoint regulation, DNA repair, and activation of MYC targets. These pathways represent central mechanisms that support uncontrolled tumor growth, maintain replicative capacity, and confer resistance to cellular stress. The enrichment of these programs suggests that MYBL2-high tumors may represent a transcriptionally active malignant state characterized by heightened cell-cycle dependence. Importantly, these findings should be interpreted as associations derived from transcriptomic analyses rather than as direct evidence of MYBL2-mediated tumor promotion. Functional experiments, including MYBL2 knockdown or overexpression models, will be needed to determine whether MYBL2 drives LSCC progression or primarily reflects an underlying proliferative phenotype.

A major strength of this study is its integration of bulk and single-cell transcriptomic evidence to overcome each approach's limitations. Bulk RNA-seq provides large-scale molecular information and enables robust statistical analysis across multiple cohorts; however, it averages signals across heterogeneous cellular populations and may obscure rare malignant states. scRNA-seq enables characterization of tumor cellular diversity and identification of malignant epithelial populations with distinct transcriptional profiles [16,32]. By integrating high-CNV malignant epithelial markers from single-cell analysis with bulk differential expression and WGCNA-associated genes, this study aimed to prioritize biomarkers that reflect tumor-specific biology rather than differences between heterogeneous tissue samples.

Single-cell findings also indicated that MYBL2-associated malignant states were not consistent across tumor epithelial populations. Among the malignant epithelial subclusters with high copy number variation (High-CNV), Cluster 0 exhibited the highest average MYBL2 expression, while Cluster 8 contained the largest proportion of MYBL2-expressing cells. Although pathway activity differences between these clusters were modest, both populations demonstrated enrichment of MYBL2-associated transcriptional signatures in bulk LSCC cohorts. This consistency across different platforms suggests that MYBL2 may reflect a broader malignant epithelial program rather than being limited to a specific cellular subgroup. Previous single-cell investigations in head and neck cancer have revealed considerable intratumoral heterogeneity, with distinct malignant epithelial states linked to varied proliferative, differentiation, and microenvironmental interactions [33]. Consequently, MYBL2 expression could serve as a molecular indicator of particular malignant cell states that contribute to tumor complexity.

The pseudotime analysis provided additional insight into the dynamic nature of MYBL2-associated malignant states. Although we observed increased MYBL2 expression across the inferred transcriptional ordering, these results should not be interpreted as direct evidence of tumor evolution or temporal progression. Computational trajectory analyses infer relationships among transcriptional states but cannot establish true biological lineage without experimental validation. Nevertheless, MYBL2 variation across malignant epithelial states suggests that tumor cells with different proliferative programs may coexist within the same tumor environment. Future studies integrating longitudinal sampling, spatial transcriptomics, and functional validation could clarify whether MYBL2-high states represent transient cellular adaptations or stable malignant phenotypes.

Beyond tumor-intrinsic characteristics, this study also examined the relationship between MYBL2 expression and the tumor microenvironment. Initial analyses indicated lower immune and stromal signature scores in MYBL2-high tumors; however, most associations were attenuated after adjusting for tumor purity. This finding underscores an important consideration in bulk transcriptomic studies: apparent immune differences may partially reflect variation in tumor cellular composition rather than true biological exclusion of immune populations. After controlling for purity, associations with selected immune populations, including NK T cells, regulatory T cells, and immature B cells, remained significant, suggesting that MYBL2-associated malignant states may still influence specific immune interactions. Similar observations have emphasized the importance of integrating tumor purity estimation with multidimensional immune profiling when interpreting immune signatures from bulk RNA-seq data [24,25].

Cell–cell communication analysis further suggested potential interactions between MYBL2-associated malignant epithelial populations and surrounding microenvironmental cells. Predicted MDK-, MIF-, and SPP1-related signaling pathways are biologically plausible, as these pathways have been implicated in tumor growth, immune modulation, extracellular matrix remodeling, and cancer-associated inflammation. However, these interactions are computational predictions based on ligand–receptor databases and should be considered hypothesis-generating rather than definitive evidence. Experimental validation using spatial transcriptomics, multiplex imaging, or protein-level assays is needed to determine whether MYBL2-high malignant cells actively shape their surrounding microenvironment.

The ML component of this study further supports MYBL2 as a robust candidate transcriptomic biomarker. Both SVM and RF models successfully classified tumor and normal samples in the independent external cohort, with SVM performing better. Machine learning methodologies have increasingly been adopted in cancer biomarker discovery because they can recognize intricate molecular patterns that traditional statistical techniques might overlook. Prior research has evidenced the utility of machine learning models for predicting clinical outcomes and identifying molecular signatures in LSCC and other oncological contexts [1]. Nevertheless, model performance depends strongly on cohort composition, sample size, and the quality of external validation. Therefore, additional validation using larger multicenter datasets will be essential to determine the generalizability of MYBL2-based prediction models.

Several limitations should be acknowledged. First, this study relied primarily on retrospective public datasets, which may differ in sequencing platforms, sample processing, and clinical characteristics. Although we performed external validation, prospective clinical cohorts are needed to confirm MYBL2′s translational value. Second, the single-cell analysis was based on only three LSCC tumors, limiting the ability to capture the full spectrum of malignant cellular diversity. Third, clinical variables such as tumor grade, HPV status, treatment response, and survival outcomes were unavailable or incomplete, limiting interpretation of associations between MYBL2 and clinical characteristics and outcomes. Additionally, inferCNV-based identification of malignant cells depends on reference population selection and may introduce computational bias. Finally, we estimated immune infiltration using transcriptomic signatures rather than direct experimental measurement. HPA/CPTAC data were insufficient to validate MYBL2 as an LSCC-specific protein, supporting its classification as a candidate transcriptomic biomarker.

In conclusion, this study identifies MYBL2 as a promising transcriptomic biomarker associated with malignant epithelial states and proliferative programs in LSCC. Integrating bulk RNA-seq, single-cell analysis, network-based approaches, and ML provides a comprehensive framework for biomarker discovery in heterogeneous tumors. Although MYBL2 requires further biological and clinical validation, its consistent association with cell-cycle regulation, malignant subpopulations, and tumor transcriptional programs highlights its potential relevance to future LSCC molecular classification and precision oncology strategies.

## Conclusion

This study identifies MYBL2 as a candidate transcriptomic biomarker associated with malignant epithelial states and proliferative pathways in LSCC. MYBL2 demonstrated consistent tumor–normal discrimination across independent cohorts and was linked to proliferation-related transcriptional pathways and malignant epithelial subpopulations. Further biological and clinical validation in clinically relevant cohorts is needed to determine its potential role in LSCC molecular classification and precision oncology.

## Supplementary

Supplementary information associated with this article includes Figures S1–S5 and Tables S1–S2, comprising sensitivity analyses, random-sampling stability assessment, evaluation of the five-gene panel, benchmarking of MYBL2 against established LSCC-related biomarkers, and tumor-purity-adjusted immune-association results.

## Declaration of generative artificial intelligence (AI) use

AI-assisted tools were used for language editing and to improve the manuscript's readability; the authors reviewed and approved all analyses, interpretations, and final content.

## Funding

The authors received no specific funding for this work.

## Ethics approval and consent to participate

Not applicable.

## CRediT authorship contribution statement

**Tahmineh Mehrabi:** Writing – original draft, Visualization, Investigation, Formal analysis, Data curation, Conceptualization, Software. **Marzie Isavi:** Formal analysis, Methodology, Writing – review & editing. **Ali Gholami:** Formal analysis, Investigation, Writing – review & editing. **Amir Ali Hamidieh:** Formal analysis, Writing – review & editing, Visualization. **Roozbeh Heidarzadeh-Pilehrood:** Writing – review & editing, Writing – original draft, Visualization, Supervision, Project administration, Methodology, Formal analysis, Conceptualization, Validation.

## Declaration of competing interest

The authors declare that they have no known competing financial interests or personal relationships that could have appeared to influence the work reported in this paper.

## Data Availability

The datasets analyzed in this study are publicly available in the Gene Expression Omnibus (GEO) database under accession numbers GSE127165, GSE142083, GSE130605, and GSE206332. This study generated no new datasets.
