# Supplementary material for: Integrating single-cell and bulk RNA sequencing with ML algorithms identified MYBL2 as a candidate transcriptomic biomarker for diagnosing laryngeal squamous cell carcinoma (LSCC)
Source: Transl Oncol. 2026 Nov 1;74:103095. doi: 10.1016/j.tranon.2026.103095 (PMC13742195; doi:10.1016/j.tranon.2026.103095)
Supplement: Supplementary file 1 [file mmc1.docx]

**Integrating single-cell and bulk RNA sequencing with ML algorithms identified MYBL2 as a candidate transcriptomic biomarker for diagnosing laryngeal squamous cell carcinoma (LSCC)**

**Supplementary Information**

# Contents

- Figure S1. Stability of tumor–normal discriminatory performance across random sampling seeds
- Figure S2. WGCNA soft-thresholding sensitivity analysis
- Figure S3. Single-cell marker-definition sensitivity analysis
- Figure S4. Tumor–normal performance of the five LASSO-selected genes and five-gene panel
- Figure S5. Transcript-level benchmarking of MYBL2 against LSCC-related markers
- Table S1. Performance of the five LASSO-selected genes and five-gene panel
- Table S2. Tumor-purity-adjusted immune-cell associations

**Figure S1. Stability of tumor–normal discriminatory performance across alternative random-sampling seeds.**

Area under the receiver operating characteristic curve (AUROC) values for MYBL2 in the training and internal validation cohorts, together with external-cohort AUROCs of the random forest (RF) and support vector machine (SVM) classifiers, are shown for sampling seeds 1, 123, 456, and 999. For each repetition, the specified seed controlled class balancing and the approximately 7:3 training–validation allocation, whereas model-fitting randomness was fixed at seed 123. The development cohort contained 219 individual bulk-tissue samples; balancing retained 218 samples, excluding only one. The analytical unit was the individual bulk-tissue sample: training *n* = 154 (77 tumors and 77 normal samples), validation *n* = 64 (32 tumors and 32 normal samples), and independent external GSE130605 *n* = 100 (50 tumors and 50 normal samples). The dashed line denotes the operational AUROC threshold of 0.95, and cross-seed comparisons are descriptive. In the primary seed-123 analysis, SVM showed a higher external AUROC than RF (0.9624 vs. 0.9100; difference = 0.0524; paired two-sided DeLong Z = −2.7718, *P* = 0.0056); this is reported as an empirical model-specific difference rather than evidence of a biological mechanism.

**Figure S2. Sensitivity analysis of WGCNA results across soft-thresholding powers (β = 12–20).**

Weighted gene co-expression network analysis (WGCNA) was repeated at soft-thresholding powers β = 12, 14, 16, 18, and 20 with all other settings fixed. (A) Strongest positive Pearson correlation between a module eigengene and laryngeal squamous cell carcinoma (LSCC) tumor status. (B) Number of genes in the strongest positively tumor-associated module. (C) Jaccard similarity between the feature genes selected at each β and those selected in the reference β = 20 analysis. (D) MYBL2 retention status. Each point represents one tested β value; the panels summarize a parameter-sensitivity analysis, and no separate inferential hypothesis test was applied across β values. The analytical unit was the individual training-cohort bulk-tissue sample (*n* = 154; 77 tumors and 77 normal samples). The primary β = 20 network had a signed scale-free topology fit of R² = 0.8918. The strongest positive module–tumor association remained stable, module size was not maximal at β = 20, and MYBL2 was retained at every tested power, arguing against systematic over-aggregation specific to β = 20.

**Figure S3. Sensitivity analysis of High-CNV malignant epithelial marker selection under alternative single-cell marker definitions.**

High copy-number variation (High-CNV) malignant epithelial markers from single-cell RNA sequencing (scRNA-seq) were identified using average log2 fold-change (avg_log2FC) thresholds of 1.5 (permissive), 2.0 (primary), and 2.5 (stringent), while the minimum fraction of cells expressing the gene (min.pct) was fixed at 0.50 and the Bonferroni-adjusted P-value threshold at < 0.001. Seurat FindAllMarkers comparisons used the Wilcoxon rank-sum test. The three definitions yielded 1,249, 707, and 419 High-CNV markers, respectively; 32, 27, and 23 genes remained after intersection with bulk upregulated differentially expressed genes (DEGs), followed by 21, 16, and 13 genes after intersection with weighted gene co-expression network analysis (WGCNA)-derived tumor-associated genes. MYBL2 was retained under all definitions. Data were obtained from three independent LSCC tumors (GSM6251294, GSM6251297, and GSM6251300); the individual cell was the computational analytical unit, whereas the patient tumor was the biological sampling unit. Histological grade and human papillomavirus status were unavailable. These findings are exploratory, and the sensitivity analysis does not eliminate uncertainty or potential overfitting arising from sequential feature filtering.

**Figure S4. Tumor–normal discriminatory performance of the five LASSO-selected genes and five-gene logistic-regression panel.**

Receiver operating characteristic (ROC) curves are shown for (A) the training cohort, (B) the internal validation cohort, and (C) the independent GSE130605 cohort. The five genes retained by least absolute shrinkage and selection operator (LASSO) analysis—MYBL2, KRT17, PTHLH, UCHL1, and ODC1—were evaluated individually and as a multivariable logistic-regression panel. The panel was fitted only in the training cohort after standardization with training-derived parameters and was applied to the validation and external cohorts without retraining. The analytical unit was the individual bulk-tissue sample: training *n* = 154 (77 tumors and 77 normal samples), validation *n* = 64 (32 tumors and 32 normal samples), and external *n* = 100 (50 tumors and 50 normal samples). An AUROC ≥ 0.95 was used as a stringent operational criterion for candidate prioritization in both the training and validation cohorts. AUROCs with 95% confidence intervals are reported in Table S1; panel-versus-MYBL2 comparisons used paired two-sided DeLong tests. The diagonal dashed line indicates AUROC = 0.50. These results quantify tumor–normal discrimination and do not establish clinical diagnostic applicability.

**Figure S5. Transcript-level benchmarking of MYBL2 against established LSCC-related markers.**

Tumor–normal receiver operating characteristic (ROC) curves compare MYBL2 with three established or previously reported laryngeal squamous cell carcinoma (LSCC)-related markers—p16 (CDKN2A), EGFR, and Ki-67 (MKI67)—in (A) the training cohort, (B) the internal validation cohort, and (C) the independent GSE130605 cohort. The analytical unit was the individual bulk-tissue sample: training *n* = 154 (77 tumors and 77 normal samples), validation *n* = 64 (32 tumors and 32 normal samples), and external *n* = 100 (50 tumors and 50 normal samples). ROC direction was determined in the training cohort and fixed in the validation and external cohorts. AUROCs with 95% confidence intervals were calculated, and MYBL2 was compared with each marker within each cohort using paired two-sided DeLong tests. The diagonal dashed line indicates AUROC = 0.50. Because all comparisons used transcript abundance, they provide benchmarking context and do not demonstrate clinical superiority over established protein-level biomarkers.

# Table S1. Tumor–normal discriminatory performance of the five LASSO-selected genes and five-gene logistic-regression panel across three cohorts.

| **Cohort** | **Model** | **AUROC (95% CI)** | **Δ AUROC (panel − MYBL2)** | **DeLong *P*-value** |
| --- | --- | --- | --- | --- |
| Training n = 154 (77 normal / 77 tumor) | MYBL2 | 0.9712 (0.9433–0.9990) | - | - |
| Training n = 154 (77 normal / 77 tumor) | KRT17 | 0.9477 (0.9068–0.9886) | - | - |
| Training n = 154 (77 normal / 77 tumor) | UCHL1 | 0.8384 (0.7733–0.9036) | - | - |
| Training n = 154 (77 normal / 77 tumor) | PTHLH | 0.9054 (0.8583–0.9525) | - | - |
| Training n = 154 (77 normal / 77 tumor) | ODC1 | 0.9015 (0.8529–0.9501) | - | - |
| Training n = 154 (77 normal / 77 tumor) | Five-gene panel | 0.9789 (0.9567–1.0000) | 0.0078 | 0.2558 |
| Validation n = 64 (32 normal / 32 tumor) | MYBL2 | 0.9658 (0.9292–1.0000) | - | - |
| Validation n = 64 (32 normal / 32 tumor) | KRT17 | 0.9131 (0.8359–0.9903) | - | - |
| Validation n = 64 (32 normal / 32 tumor) | UCHL1 | 0.8018 (0.6849–0.9186) | - | - |
| Validation n = 64 (32 normal / 32 tumor) | PTHLH | 0.9014 (0.8183–0.9845) | - | - |
| Validation n = 64 (32 normal / 32 tumor) | ODC1 | 0.7656 (0.6457–0.8856) | - | - |
| Validation n = 64 (32 normal / 32 tumor) | Five-gene panel | 0.9512 (0.9033–0.9990) | -0.0146 | 0.4047 |

# Table S1 (continued). Independent external GSE130605 cohort.

| **Cohort** | **Model** | **AUROC (95% CI)** | **Δ AUROC (panel − MYBL2)** | **DeLong *P*-value** |
| --- | --- | --- | --- | --- |
| External GSE130605 n = 100 (50 normal / 50 tumor) | MYBL2 | 0.9624 (0.9239–1.0000) | - | - |
| External GSE130605 n = 100 (50 normal / 50 tumor) | KRT17 | 0.9280 (0.8777–0.9783) | - | - |
| External GSE130605 n = 100 (50 normal / 50 tumor) | UCHL1 | 0.8248 (0.7353–0.9143) | - | - |
| External GSE130605 n = 100 (50 normal / 50 tumor) | PTHLH | 0.9384 (0.8948–0.9820) | - | - |
| External GSE130605 n = 100 (50 normal / 50 tumor) | ODC1 | 0.9108 (0.8499–0.9717) | - | - |
| External GSE130605 n = 100 (50 normal / 50 tumor) | Five-gene panel | 0.9768 (0.9525–1.0000) | 0.0144 | 0.3135 |

# Table S2. Tumor-purity-adjusted associations between MYBL2 expression and immune-cell enrichment.

| **Immune-cell signature** | ***n*** | **Primary partial ρ** | **Primary *P*-value** | **Primary BH-FDR** | **Sensitivity partial ρ** | **Sensitivity *P*-value** | **Sensitivity BH-FDR** |
| --- | --- | --- | --- | --- | --- | --- | --- |
| Natural killer T cell | 109 | 0.3677 | 9.03 × 10⁻⁵ | 0.0025 | 0.3669 | 1.01 × 10⁻⁴ | 0.0028 |
| Regulatory T cell | 109 | 0.3367 | 3.66 × 10⁻⁴ | 0.0051 | 0.3354 | 4.13 × 10⁻⁴ | 0.0058 |
| Immature B cell | 109 | −0.2725 | 0.00433 | 0.0405 | −0.2737 | 0.00434 | 0.0405 |
